# Consuming Patients’ Days: Time Spent on Ambulatory Appointments by People With Cancer

**DOI:** 10.1093/oncolo/oyae016

**Published:** 2024-02-10

**Authors:** Sana Kagalwalla, Alexander K Tsai, Manju George, Anna Waldock, Sydney Davis, Patricia Jewett, Rachel I Vogel, Ishani Ganguli, Christopher Booth, Stacie B Dusetzina, Gabrielle B Rocque, Anne H Blaes, Arjun Gupta

**Affiliations:** University of Minnesota, Minneapolis, MN, USA; University of Minnesota, Minneapolis, MN, USA; Paltown Development Foundation/COLONTOWN, Crownsville, MD, USA; University of Minnesota, Minneapolis, MN, USA; University of Minnesota, Minneapolis, MN, USA; University of Minnesota, Minneapolis, MN, USA; University of Minnesota, Minneapolis, MN, USA; Division of General Internal Medicine and Primary Care, Brigham and Women’s Hospital, Boston, MA, USA; Department of Oncology, Queen’s University, Kingston, Canada; Department of Health Policy, Vanderbilt University Medical Center, Nashville, TN, USA; Vanderbilt-Ingram Cancer Center, Nashville, TN, USA; Division of Hematology and Oncology, University of Alabama at Birmingham, Birmingham, AL, USA; University of Minnesota, Minneapolis, MN, USA; University of Minnesota, Minneapolis, MN, USA

**Keywords:** contact days, time toxicity, time burdens, logistic toxicity, ambulatory appointments

## Abstract

**Background:**

In qualitative work, patients report that seemingly short trips to clinic (eg, a supposed 10-minute blood draw) often turn into “all-day affairs.” We sought to quantify the time patients with cancer spend attending ambulatory appointments.

**Methods:**

We conducted a retrospective study of patients scheduled for oncology-related ambulatory care (eg, labs, imaging, procedures, infusions, and clinician visits) at an academic cancer center over 1 week. The primary exposure was the ambulatory service type(s) (eg, clinician visit only, labs and infusion, etc.). We used Real-Time Location System badge data to calculate clinic times and estimated round-trip travel times and parking times. We calculated and summarized clinic and total (clinic + travel + parking) times for ambulatory service types.

**Results:**

We included 435 patients. Across all service day type(s), the median (IQR) clinic time was 119 (78-202) minutes. The estimated median (IQR) round-trip driving distance and travel time was 34 (17-49) miles and 50 (36-68) minutes. The median (IQR) parking time was 14 (12-15) minutes. Overall, the median (IQR) total time was 197 (143-287) minutes. The median total times for specific service type(s) included: 99 minutes for lab-only, 144 minutes for clinician visit only, and 278 minutes for labs, clinician visit, and infusion.

**Conclusion:**

Patients often spent several hours pursuing ambulatory cancer care on a given day. Accounting for opportunity time costs and the coordination of activities around ambulatory care, these results highlight the substantial time burdens of cancer care, and support the notion that many days with ambulatory health care contact may represent “lost days.”

## Teaser

This study used real-time location system and geolocation data to assess how much time (in minutes) patients spent on attending clinic appointments. Even seemingly short appointments can take up hours of patients' days, supporting the notion that that day may indeed represent a ''lost day''.

**Implications for Practice**

In prior work, patients reported that seemingly short clinic trips (eg, a supposed 10-minute blood draw) often turn into “all-day affairs.” In this study, using real-time location system and geolocation data, we found that patients often spent several hours home-to-home in pursuing routine ambulatory cancer care on a given day. Accounting for opportunity costs and the coordination of activities around ambulatory care, these results support the notion that any ambulatory health care contact can take up hours of patients’ time on that day, and that day may indeed represent a “lost day.”

## Introduction

Days spent receiving health care can place major time burdens on patients with cancer. There is growing recognition of the importance of measuring “health care contact days,” or “contact days”—days when patients must leave their home to seek health care, regardless of the reason and length of time spent that day—as a measure of how much of a person’s life is consumed by health care.^1,2^ Contact days are used as a measure of care efficiency in the real world and as a clinical trial outcome.^3-5^ Patients with advanced solid tumors can spend 20%-25% of their days alive with health care contact.^6-8^ A majority of these contact days are ambulatory, requiring patients and accompanying care partners to undertake frequent and burdensome back-and-forth trips to clinic.^7^

While the oncology community considers overnight stays in a health care facility (inpatient contact days) as “bad days,” days with only ambulatory health care contact are not routinely captured, reported, or considered burdensome in oncology clinical trials.^9,10^ In qualitative studies, patients with cancer have commented on how seemingly simple and purportedly short clinic appointments can turn into all-day affairs for themselves and care partners, and how they frequently are required to plan their whole day around a single clinic appointment.^1,11^ This is partly because of the recognition that in comparison to inpatient contact days that take up a whole day (except for admission and discharge days), ambulatory contact days take up only part of a patient’s day. Only limited data exist on how much time patients with cancer spend in a day when attending ambulatory appointments—a single-center observational study shadowing 39 patients with advanced breast cancer in 2016-2017 found that patients spent an average of 220 minutes in the clinic.^12^ In a parallel survey, patients reported spending an average of 156 minutes in the clinic.^12^ These results are limited by a small number of direct observations and are subject to recall bias. The duration of oncology appointments likely varies considerably based on patient complexity, the number of ambulatory services received, travel time, and other factors. To date, however, the time required to complete ambulatory cancer appointments has not been comprehensively quantified.

There is a critical need to objectively measure time spent by patients with cancer receiving ambulatory care in contemporary practice, specifically accounting for the amount and complexity of services provided and patient travel time. These data could inform the inclusion of days with ambulatory cancer care in the measure of overall contact days, while also providing meaningful baseline data and identifying potential opportunities to improve the efficiency of the health care system. Therefore, we sought to measure the home-to-home time (including clinic and travel time) spent by patients with cancer when pursuing ambulatory care at an urban cancer center. An additional objective was to examine the time spent when pursuing specific type(s) of ambulatory services to identify differences in time required between, for example, a lab appointment alone compared to a more complex clinician examination with subsequent infusion.

## Methods

### Study Design and Population

We conducted a retrospective study of adult patients with cancer scheduled for any of the following ambulatory service(s): laboratory testing, imaging, procedures, infusions, and medical oncology clinician visits. The study setting was within the MHealth Fairview, University of Minnesota Masonic Cancer Center clinic site, in Minneapolis, MN—an urban National Cancer Institute-designated Comprehensive Cancer Center—during a randomly selected 5-day work week in January 2023. The evaluation was limited to 1 week as the data for each patient visit at the clinic needed to be extracted and compared to the documentation in electronic medical records manually. We identified eligible patient encounters by reviewing oncology-specific laboratory, imaging, procedure, infusion, and clinician schedules. All appointments, including laboratory testing, were scheduled and did not include more unpredictable “walk-in” visits. We initially identified 526 encounters among 488 distinct patients. We excluded patients with nonmalignant diseases (eg, sickle cell disease or aplastic anemia who were receiving infusions in the oncology center) and those who opted out of their medical data being used for research purposes. The study flowchart is presented in Supplementary Fig. S1. The University of Minnesota Institutional Review Board reviewed the study protocol and determined that informed consent from participants was not required.

### Measures

Our primary outcome of interest was the home-to-home time associated with ambulatory cancer care as the sum of clinic time, travel time, and parking time. A secondary outcome was time spent within the clinic on specific ambulatory services. In the clinic, to optimize clinical workflow, it is standard-of-care to provide patients with a real-time location system (RTLS) badge (Midmark, Inc., Supplementary Fig. S2) at clinic entry. Patients are instructed to remove and return the badges upon final exit from the clinic premises. All clinic staff are also encouraged to wear assigned RTLS badges throughout their time in the clinic. The badges update and record wearer locations every 3 seconds via infrared pulses detected by environmental sensors (receivers) located throughout the clinic. We reviewed the RTLS badge data to calculate the time spent by patients in the clinic. We reviewed the time spent within a location in the clinic for services the patient received as documented in the electronic medical record. When an inordinate amount of time was spent on a clinical activity (eg, >300 minutes in the exam room), RTLS data were further evaluated to determine whether a badge error occurred or whether a badge was left behind at a location. In instances (*n* = 3) where we could confidently conclude that data were inaccurate, we excluded the clinical activity time. If a patient had a clinician visit and an infusion per the electronic medical record but only spent time in the infusion center, we confirmed that the clinician conducted the visit during infusion via documentation in the electronic medical record.

Travel (driving) times to and from the cancer center (zip code 55455) were estimated based on home zip codes as reported in medical records using an SAS function integrating Google Maps. We used a standardized day and time of Thursday at 3 p.m. for each patient. We doubled the estimated one-way travel time to estimate round-trip travel time. We estimated parking times by retracing the hypothetical paths of patients between the clinic entry/exit and the main patient garage. Two study team members (S.K. and A.K.T.) independently walked from 4 ends of each of the 6 floors of the main patient-parking garage (a total of 24 data points per team member) to clinic entry, and from the clinic exit to the garage during the same week in January 2023. We calculated parking times by calculating the median sum of the toward-clinic and from-clinic parking times for each garage point. Of note, the parking times did not include the time patients spent trying to find a parking spot in the garage (garage entry to parking their vehicle).

We additionally extracted patient sociodemographic and clinical information from the electronic medical record, including age, sex, ethnicity, race, home zip code, and site of primary cancer for subgroup comparisons.

### Data Analysis

We identified the type(s) of ambulatory services (eg, clinician visit, labs, imaging, infusion, or combinations) a patient completed on a given day with ambulatory care. For example, if a patient had only a clinician visit that day, it was classified as a “clinician visit.” If a patient had labs before or after a clinician visit, it was classified as “labs + clinician visit.” To calculate clinic time, we extracted the time stamps measured by the RTLS badges for each patient as they progressed through designated areas in the clinic (eg, lobby entry/exit, exam room entry/exit, and infusion center entry/exit). We calculated time spent in a designated area for each patient. Medians and interquartile ranges (IQR) were calculated for time in clinic and total home-to-home (clinic + travel + parking) time. We summarized clinic times and total times (primary outcomes) for the different ambulatory appointment types and visualized them using box and whiskers plots. We evaluated and compared clinic times and total times by sociodemographic factors (age, sex, race/ethnicity) and the primary site of cancer using a Mann-Whitney test when comparing 2 groups and Kruskal-Wallis test when comparing more than 2 groups. We used Microsoft Excel and Prism (Version 10.0.02, Graphpad) for analysis and figures, respectively.

## Results

We included 435 unique patients in the study. The median age of patients was 64 years (IQR 53-72 years), 54% were female, and 82% identified as white. The most common primary cancer sites/types were breast (21%) and hematologic cancers (18%). Table 1 presents baseline sociodemographic and clinical characteristics of the 435 patients.

**Table 1.** Sociodemographic and clinical characteristics of patients (*n* = 435 patients).

Variable	Number of patients	Percentage of total patients
Age (years)		
18-44	64	14.7
45-64	169	38.9
65-74	135	31.0
≥75	67	15.4
Sex		
Female	234	53.8
Male	201	46.2
Self-reported race		
Asian	18	4.1
Black	40	9.2
Other or unknown	22	5.1
White	355	81.6
Ethnicity		
Hispanic or Latino	9	2.1
Not Hispanic or Latino	373	85.7
Choose not to answer	53	12.2
Primary cancer site		
Breast	89	20.5
Central nervous system	21	4.8
Gastrointestinal	48	11.0
Genitourinary/reproductive	77	17.7
Head/neck	19	4.4
Hematologic	80	18.4
Sarcoma	20	4.6
Skin	13	3.0
Thoracic	43	9.9
Unknown primary/other	25	5.7

Of the 435 days with ambulatory care, 159 (37%) involved a single service, 195 (45%) involved 2 services, and 72 (17%) involved 3 services performed. Nine (2%) of days had 4 or more services performed. The most common service type(s) were clinician visit only (*n* = 112, 26%), labs and clinician visit (*n* = 77, 18%), and labs, clinician visit, and infusion (*n* = 55, 13%).

Across service type(s), the median (IQR) clinic time was 119 (78-202) minutes. The median (IQR) estimated travel time was 50 (36-68) minutes and the median (IQR) round-trip driving distance was 34 (17-49) miles. The estimated median (IQR) parking time was 14 (12-15) minutes. Overall, across service type(s), the median (IQR) total time (clinic + travel + parking times) was 197 (143-287) minutes.

**Figure 1.**
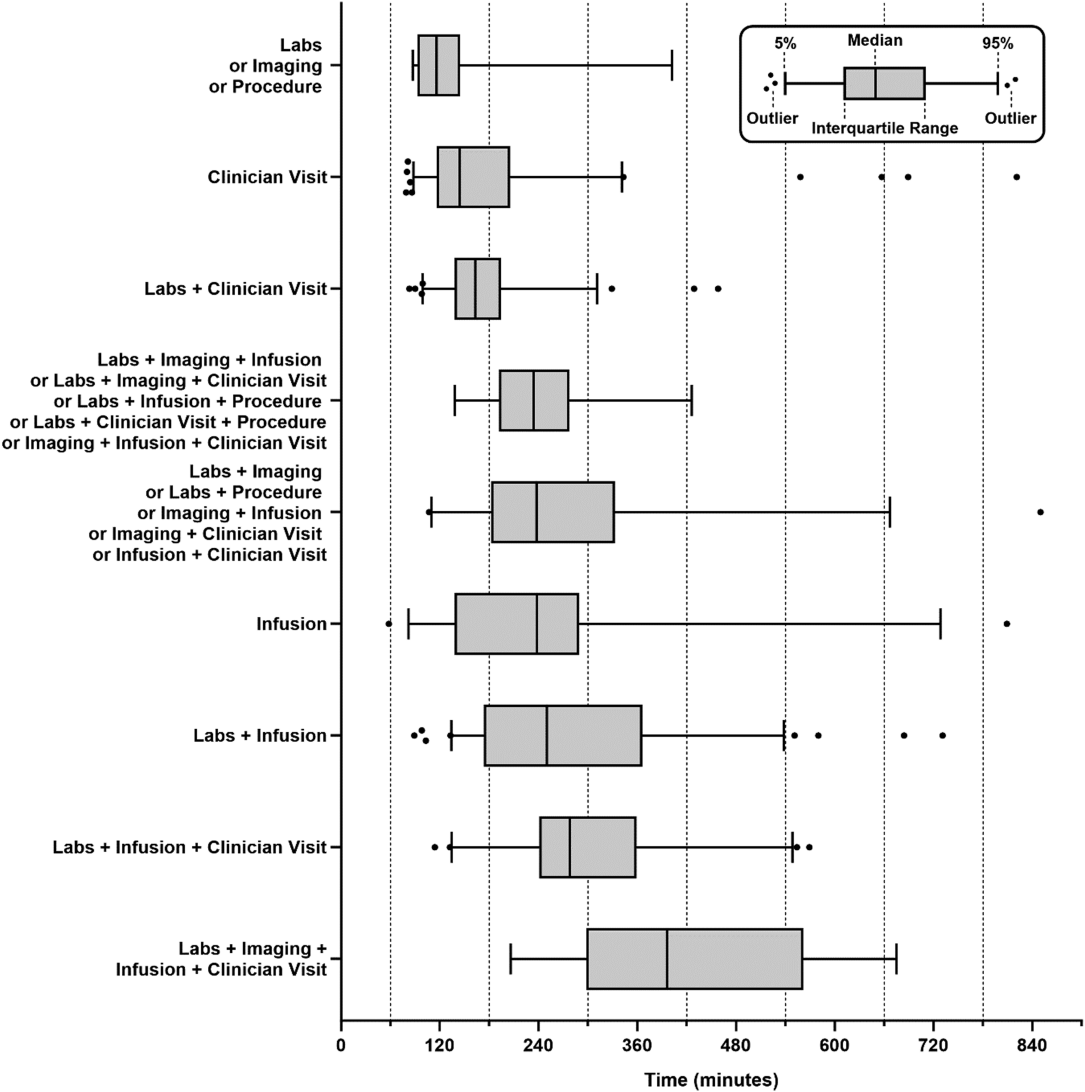
Total time spent on days with ambulatory care by service type(s) received that day. Encounters were grouped based on service type(s) received and total time was quantified and represented with median, IQR (box), 5th and 95th percentiles (whiskers), and outliers (filled circles). Groups were broadly separated based on the number of activities and number of patients with smaller groups combined based on the number of activities.

**Figure 2.**
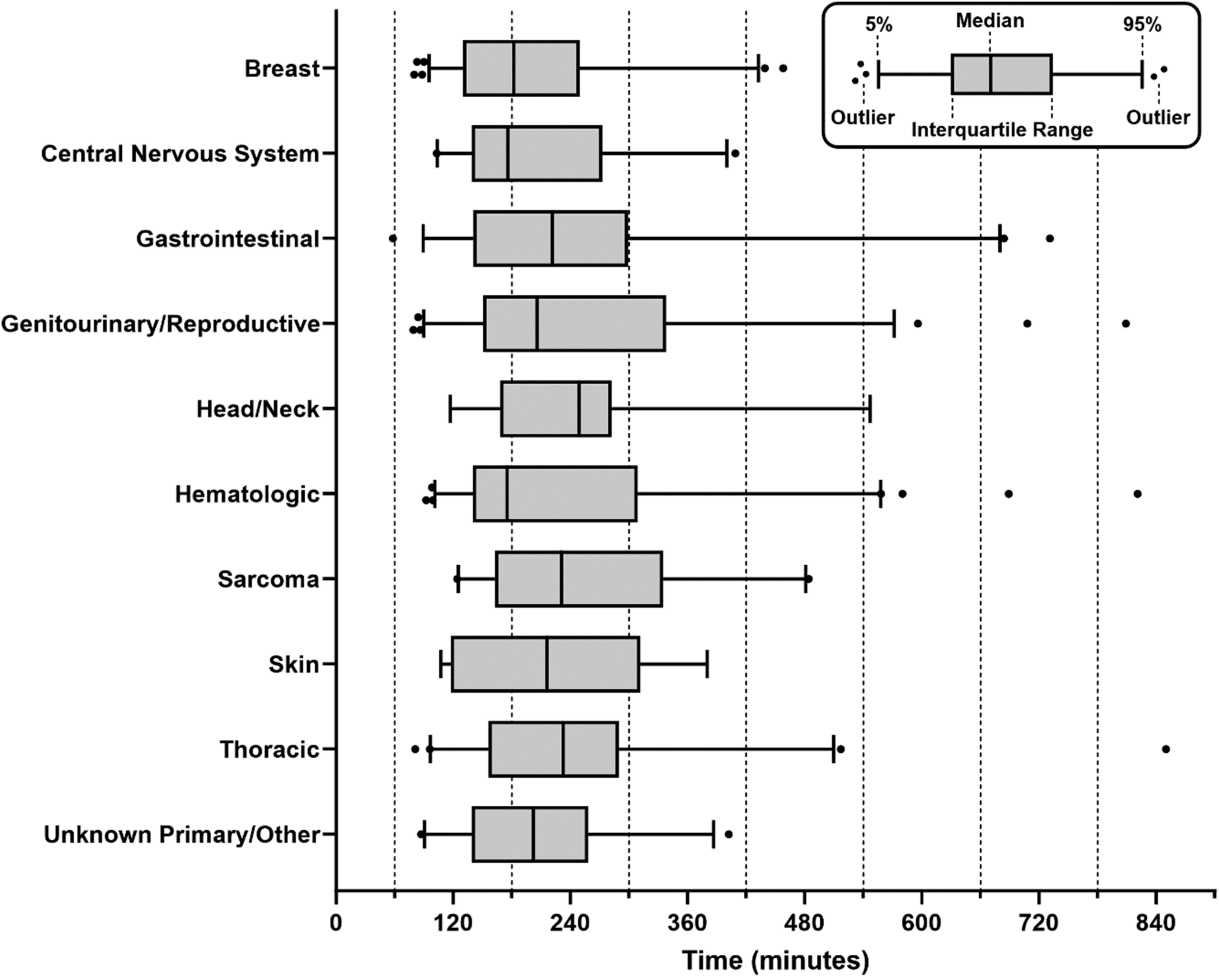
Total time spent on days with ambulatory care by site of primary cancer. Days were grouped based on the site of primary cancer and total time was quantified and represented with median, IQR (box), 5th and 95th percentiles (whiskers), and outliers (filled circles). *P* = .2147, Kruskal-Wallis test.

Clinic time and total time by ambulatory service type(s) are summarized in Table 2. Given the wide range of data points, we present the distribution of times by service type(s) using box and whiskers plots in Fig. 1. Notably, the median total times for specific service type(s) included: 99 minutes for lab-only, 144 minutes for clinician visit only, and 278 minutes for labs, clinician visit, and infusion. The 75th percentile of total times were 129 minutes for lab-only, 204 minutes for clinician visit only, and 354 minutes for labs, clinician visit, and infusion. Clinic times and total times were similar across sociodemographic characteristics and by primary cancer site (Fig. 2, Supplementary Table S1).

**Table 2.** Time spent on ambulatory encounters, by type(s) of ambulatory services received on a given day (*n* = 435).

Type of service(s)	*n*	Clinic time (minutes), median (IQR)	Total time (minutes), median (IQR)
Labs	8	30 (24-37)	99 (92-129)
Infusion	35	153 (76-210)	238 (153-284)
Clinician visit	112	77 (59-109)	144 (117-204)
Imaging	1	59 (59-59)	105 (105-105)
Procedure	3	216 (151-254)	360 (250-381)
Imaging + clinician visit	15	167 (103-209)	285 (187-316)
Infusion + clinician visit	10	220 (162-289)	282 (218-346)
Labs + clinician visit	77	90 (71-106)	163 (140-194)
Labs + infusion	89	177 (115-287)	250 (174-366)
Labs + imaging	1	186 (186-186)	242 (242-242)
Labs + procedure	2	99 (81-116)	159 (138-179)
Imaging + infusion	2	105 (85-126)	178 (166-190)
Labs + clinician visit + infusion	55	217 (170-259)	278 (244-354)
Labs + imaging + clinician visit	10	193 (113-199)	233 (195-274)
Labs + imaging + infusion	3	218 (175-297)	278 (237-352)
Labs + clinician visit + procedure	2	164 (148-179)	210 (197-222)
Imaging + infusion + clinician visit	1	166 (166-166)	236 (236-236)
Labs + imaging + infusion + clinician visit	9	277 (199-352)	396 (333-527)
Overall	435	119 (78-202)	197 (143-287)

## Discussion

In this single-center study, patients with cancer receiving routine ambulatory care on a given day at an urban academic clinic spent a median of 2 hours within the clinic and over 3 hours when also accounting for travel time. A quarter of patients coming in for labs, a clinician visit, and an infusion spent 6 hours or more, and a quarter of patients coming in for labs only spent over 2 hours on each encounter day. These data highlight how even seemingly simple and purportedly short ambulatory appointments such as blood draws can impose considerable time burdens on patients with cancer.

The time associated with cancer-related ambulatory care in this study is greater than those reported for the general adult population pursuing ambulatory care, likely due to clinical complexity and increased travel requirements. In analyses of the American Time Use Survey, adults receiving clinic-based care on a given day reported spending an average of approximately 2 hours of total time (clinic plus travel time).^13,14^ Of the 86 minutes of clinic time, only approximately 20 minutes was spent face-to-face with a clinician; in comparison, 38 minutes were spent on travel.^13^ Among patients with cancer with cancer-related outpatient clinician visits, the average face-to-face time with a clinician was 23 minutes.^15^ Our findings of longer times relative to prior broader work in all adults may be due to several factors. First, our study only included care delivered in a tertiary cancer center, which may be more complex than the average community care center, resulting in longer clinic times. Additionally, patients with cancer can require more specialized care, which may increase the time needed for care delivery. We also observed that almost two-thirds of ambulatory days in our study included multiple services, which lengthens care on a given day. Expectedly, patients receiving infusions had the longest clinic times. Though minimizing the time burden is important, cancer care—and specifically drug infusions—traditionally follows numerous sequential steps. First, patients scheduled to receive systemic cancer-directed therapy typically complete a laboratory draw. This is traditionally followed by clinician evaluation and simultaneous laboratory evaluation to determine the appropriateness of therapy administration. Finally, patients proceed to an infusion center to receive treatment. Innovations in care delivery, such as text message-based questionnaires to allow patients with normal laboratory parameters to bypass the clinician visit are being developed.^16^ Second, we estimated median travel and parking times of 50 and 14 minutes, respectively. This combined time is longer than the national average (38 minutes) for all ambulatory visits.^13^ The cancer center in this study is in a metropolitan area, where traffic and parking can be cumbersome, and add several minutes to the total time. The 50-minute driving time is greater than the median 32-minute travel time for a cancer care site among older adults in the Southeastern US, and shorter than the >2 hours of travel time for patients residing in the most rural areas of Pennsylvania.^17,18^ Overall travel times for specialized cancer care are even more burdensome—in the US, the median one-way travel time to a National Cancer Institute-designated cancer center and to academic-based care is 78 and 30 minutes, respectively.^19^ These times are even longer for Native Americans, non-urban dwellers, and residents in the South.^5,19^

The results of this study are an urgent call for the oncology community and health systems to recognize, acknowledge, and improve the time burdens that a single trip to a clinic can impose on patients and care partners. We found that patients with cancer dedicated at least 2 hours to attend even the simplest of ambulatory appointments, such as a blood draw only. A clinician visit alone took almost 2.5 hours with 75 minutes spent within the clinic, even though past data indicated that average face-to-face time with a clinician is approximately 20 minutes.^15^ Planning and receiving this ambulatory care can require patients to plan their entire day around this activity. Care partners accompany many patients, and these time losses are multiplied for them. While an extra hour spent by a patient and/or care partner by itself may seem small relative to the overall time and resources invested in managing cancer, these time losses are extremely pertinent for persons who are, for example, hourly employees or those who have less flexible work hours.^20^ More than 40% of cancer survivors who worked for pay make employment changes (eg, switching to a less demanding job, etc.) after cancer diagnosis. Those survivors most at risk for financial hardship are also less likely to have access to paid sick leave, flexible work schedules, and other accommodations, leading to higher rates of job loss.^21^ These time data can be used for sophisticated microcosting to better understand the economic impact of time losses, and the overlap between time, financial, and logistics toxicity.^21-24^ While these more nuanced techniques and methods will be applied in the coming years, it is easy to imagine that oftentimes, days with ambulatory care will represent significant time loss, and sometimes even a day’s loss at the patient level. Thus, ignoring the burdens of ambulatory care and only including inpatient days as contact days grossly underestimate the time burdens of cancer care on patients. While delivering high-quality cancer care requires patients to attend appointments in the clinic, clinicians and the cancer care delivery systems should minimize unnecessary patient trips through improved care coordination and navigation, use of home-based or telehealth care when safe, feasible, and aligned with patient preferences, and improving clinic efficiency and care access to decrease both clinic and travel times.^25,26^ There are many interventions that can address these goals. Telemedicine can significantly reduce the time, travel, and cost burden, especially for patients who live in more rural communities.^24^ Home phlebotomy and home infusion therapies should also be considered. Where possible and appropriate, oral therapies can be used in place of parenteral equivalents.^24^ To decrease travel burdens, clinicians can take advantage of facilities closer to a patient’s residence. Coordinating and bunching appointments when clinically appropriate and in line with patient wishes represent low-hanging fruit. Finally, electronic text-messaging-based triage tools can be used to determine the need for pretreatment in-person clinician evaluations, with the potential to reduce unnecessary visits.^14^

This study had limitations. First, this was a single-center study—because the RTLS is only available at one site in the health system—at a metropolitan, academic cancer center—and findings may lack generalizability as most cancer care is delivered in community-based centers. Sometimes, the logistics at community centers (eg, easier parking) may decrease time spent, but travel time could be highly variable and often greater. Second, while staff at our clinic of study are encouraged to wear RTLS badges, badge usage by clinicians is low, preventing us from capturing patients’ face-to-face time with clinicians. We thus restricted our analyses to describe the overall time in the clinic, instead of more nuanced time in lobby, time in clinic waiting room, time waiting in exam room for clinician, etc. While this information would be valuable in improving local care, the primary purpose of this study was to evaluate the total time spent by patients seeking ambulatory care on a given day. However, even without exact numbers, it is easy to recognize that for clinician visits, the actual face-to-face time with a clinician would be a fraction of the patient time spent at clinic that day. In ongoing work, we are using clinician RTLS badge data (when available) for process mapping and local quality improvement. Third, the time calculations in this study may not be exactly accurate. Excluded RTLS data could represent meaningful clinical activities. The RTLS can also not track activities at a given location, and patients could choose to be in the cancer center for longer than needed (eg, have a meal). The estimated travel times (at 3 p.m. on a weekday) and parking times (closest garage) were subject to estimation errors—we could not specifically measure each patient’s actual travel and parking times. While a valet service can save patient’s time, it also imposes financial burdens.^22,27,28^ It should also be noted that travel times for patients using public and other transportation methods to access clinic were not measured. The parking times did not include time patients spent trying to find a parking spot in the garage. Overall, our estimated travel times are likely an underestimate. Ongoing work with mobile health technology might capture true transportation-related data more accurately. Fourth, because we identified patients through medical oncology-specific scheduling lists, we did not capture care provided by other specialties (including radiation oncology) or noncancer-related care. Fifth, we did not extract data on the expected duration of each appointment type. Future work will compare the time spent on ambulatory visits with the anticipated appointment duration. Lastly, due to numbers and data availability, we were unable to meaningfully compare how times varied by important social and clinical variables, such as by use of interpreter services.

## Conclusion

Pursuing and receiving ambulatory cancer care imposes significant time burdens on patients with cancer. Accounting for travel time, there are no quick “10-minute” trips to clinic, and even the simplest clinic encounters can take up hours of patients’ time. These results support the inclusion of ambulatory contact days in the overall measure of health care contact days, and provide baseline data that point to opportunities and motivation for care delivery reform to decrease patients’ time burdens.

## Supplementary Material

Supplementary material is available at *The Oncologist* online.

oyae016_suppl_Supplementary_Figures_1-2

oyae016_suppl_Supplementary_Tables_1

## Data Availability

The data underlying this article will be shared on reasonable request to the corresponding author.

## Prior Presentation

This work was presented in abstract form at the 2023 ASCO Quality Care Symposium in Boston, MA, USA in October 2023.
