# Supplementary material for: Consuming Patients’ Days: Time Spent on Ambulatory Appointments by People With Cancer
Source: Oncologist. 2024 Feb 10;29(5):400–6. doi: 10.1093/oncolo/oyae016 (PMC11067814; doi:10.1093/oncolo/oyae016)

**Supplementary Figure 1:** Study flowchart


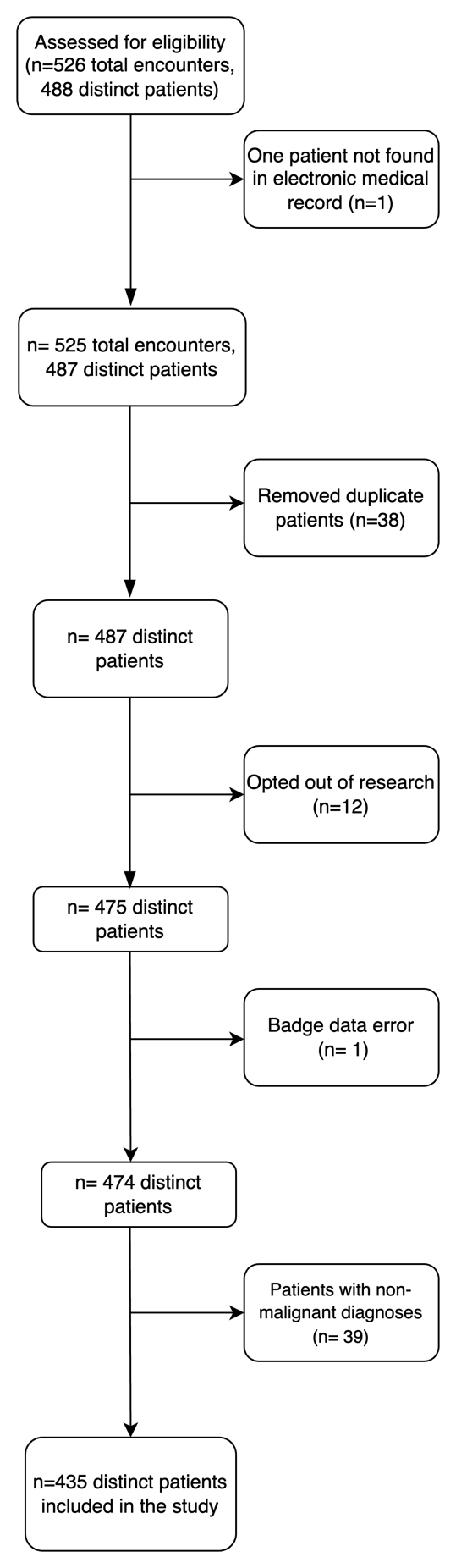


**Supplementary Figure 2:** The real-time location survey badge and how location services are used to keep track of patients and staff in clinic (all names fictional)

**
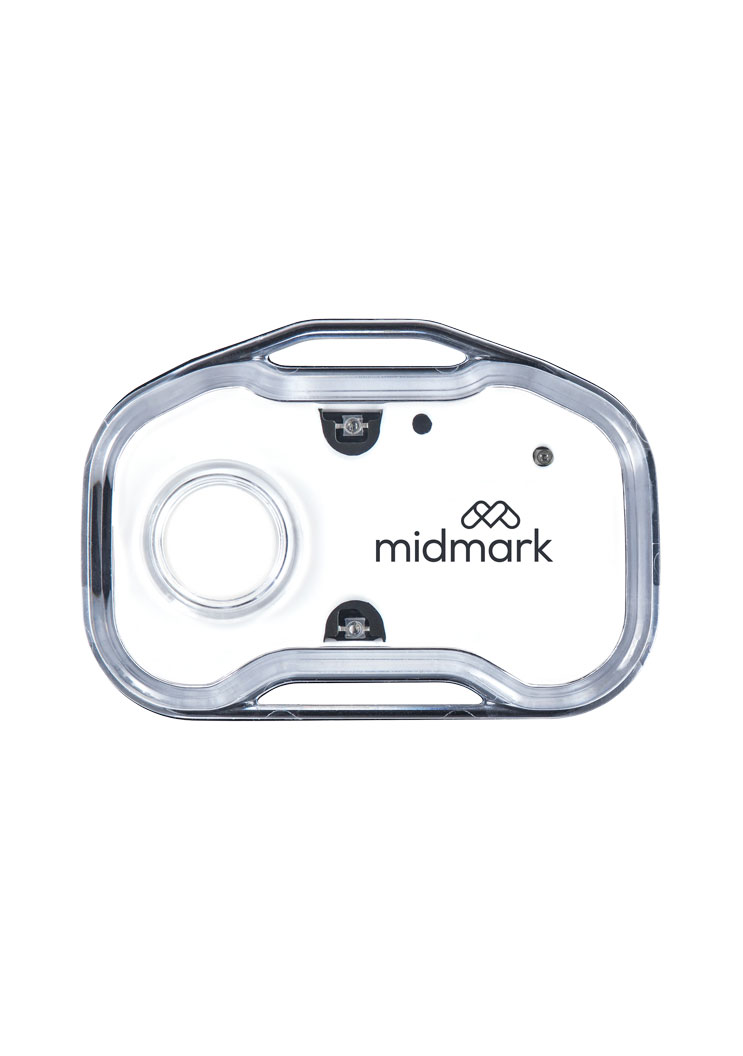
**


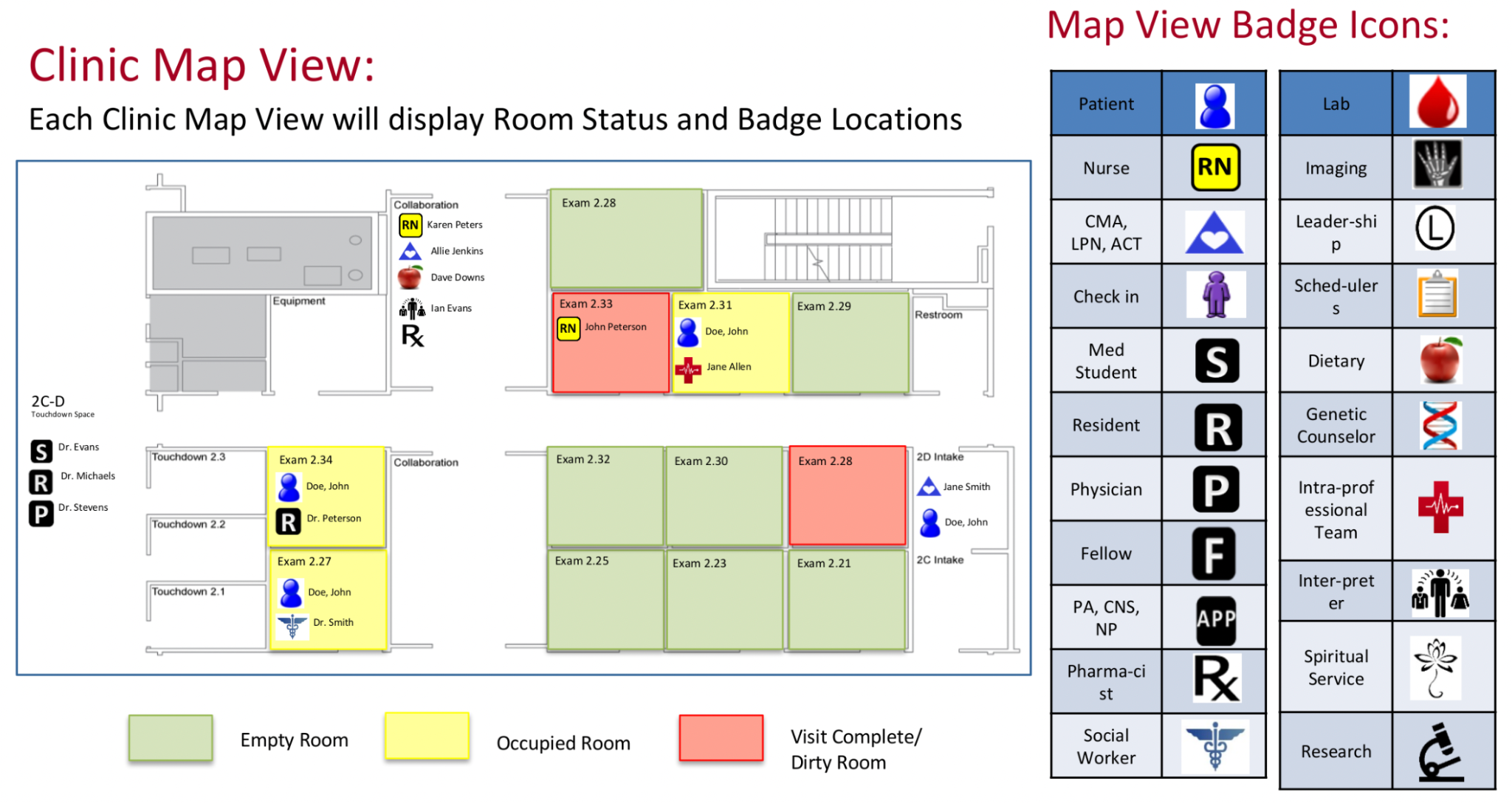

Supplement: oyae016_suppl_Supplementary_Figures_1-2 [file oyae016_suppl_supplementary_figures_1-2.docx]
