# Supplementary material for: Consuming Patients’ Days: Time Spent on Ambulatory Appointments by People With Cancer
Source: Oncologist. 2024 Feb 10;29(5):400–6. doi: 10.1093/oncolo/oyae016 (PMC11067814; doi:10.1093/oncolo/oyae016)
Supplement: oyae016_suppl_Supplementary_Tables_1 [file oyae016_suppl_supplementary_tables_1.docx]

**Supplementary Table 1:** Time spent on ambulatory encounters, by sociodemographic characteristics (n=435)

| **Variable** | **Clinic Time**  **Median (IQR), Minutes** | **p-value** | **Total Time**  **Median (IQR), Minutes** | **p-value** |
| --- | --- | --- | --- | --- |
| Age, years | | | | |
| 18-44 (n=64) | 117 (71-237) | *p*=0.7019 ^#^ | 196 (138-343) | *p*=0.3460 ^#^ |
| 45-64 (n=169) | 121 (78-203) |  | 205 (154-300) |  |
| 65-74 (n=135) | 119 (83-189) |  | 199 (144-273) |  |
| ≥75 (n=67) | 102 (68-200) |  | 178 (131-262) |  |
| Sex | | | | |
| Female (n=234) | 110 (72-199) | *p*=0.3209 ^†^ | 194 (137-295) | *p*=0.5836 ^†^ |
| Male (n=201) | 127 (81-202) |  | 200 (151-279) |  |
| Race | | | | |
| Asian (n=18) | 96 (75-202) | *p*=0.7768 ^#^ | 155 (138-266) | *p*=0.4919 ^#^ |
| Black (n=40) | 131 (81-229) |  | 185 (134-288) |  |
| Other or Unknown (n=22) | 114 (80-164) |  | 176 (141-225) |  |
| White (n=355) | 119 (76-201) |  | 204 (146-288) |  |
| Ethnicity | | | | |
| Hispanic (n=9) | 180 (79-217) | *p*=0.6671 ^#^ | 218 (136-296) | *p*=0.4888 ^#^ |
| Not Hispanic (n=373) | 119 (77-203) |  | 198 (144-289) |  |
| Chose not to answer (n=53) | 116 (78-168) |  | 179 (138-250) |  |

^#^ Kruskal-Wallis test; ^†^ Mann Whitney test

Total time = clinic time plus travel time plus parking time
